# Charting the Knowledge and Patterns of Non-Steroidal Anti-Inflammatory Drugs Usage in Hail Population, Saudi Arabia: Insights into the Adverse Effect Profile

**DOI:** 10.3390/pharmacy12010009

**Published:** 2024-01-08

**Authors:** Abdullah T. Altahini, Waled Aburas, Saud F. Aljarwan, Suliman A. Alsuwayagh, Naif F. Alqahtani, Saleh Alquwaiay, Sirajudheen Anwar

**Affiliations:** 1 College of Pharmacy, University of Hail, Hail 55476, Saudi Arabias201807668@uoh.edu.sa (S.A.); 2 Department of Pharmacology and Toxicology, College of Pharmacy, University of Hail, Hail 55476, Saudi Arabia

**Keywords:** KAP, NSAIDS, pain treatment, musculoskeletal pain, complications

## Abstract

(1) Background: It is crucial to provide safe and knowledgeable healthcare practices because no research has been performed on the knowledge and usage patterns of NSAIDs among the Hail population. (2) Method: Structured questionnaires were utilized to gather data from 399 individuals in Hail, Saudi Arabia, for the cross-sectional analysis. The study assessed participants’ knowledge regarding NSAIDs, patterns of use, reasons for use, and awareness of potential side effects. (3) Results: In the study, the gender distribution indicated that 170 participants (42.61%) were male, whereas 229 (57.39%) were female. Gender, occupation, and marital status showed non-significant associations except for menstrual cycle and joint pain, where marital status displayed significant associations (*p* > 0.001). Education and monthly income exhibited non-significant associations for all these reasons. The regression analysis demonstrated that gender played a significant role, with females having higher odds of knowledge (AOR = 1.75, 95% CI 1.10–2.88) than males. Meanwhile, >50% of the participants had knowledge of adverse events related to the use of NSAIDs, whereas 25% had no knowledge. Moreover, 59 (25.76%) participants reported discomfort with the use of NSAIDs. In addition, 50% and >75% of respondents believed that NSAIDs could induce peptic ulcers and kidney damage, respectively. (4) Conclusions: This study shed light on the knowledge and patterns of NSAIDs use in the population of Hail, Saudi Arabia. Healthcare providers and policymakers should consider these insights to develop targeted educational initiatives and healthcare interventions to promote safe and informed NSAID utilization in the region.

## 1. Introduction

Nonsteroidal anti-inflammatory drugs (NSAIDs) are a widely recognized group of medicines that possess fever-reducing, pain-relieving, and anti-inflammatory properties [1]. Approximately 30 million individuals worldwide are believed to use NSAIDs daily [2], and they are ranked among the most frequently utilized pharmaceuticals worldwide, encompassing about 5% of all prescribed and over-the-counter (OTC) medications [1,3]. From the ancient utilization of willow leaves containing salicylates to the more recent fluctuations in the popularity of highly selective cyclooxygenase-2 (COX-2) inhibitors and the emergence of the latest dual-acting anti-inflammatory compounds, there has been a continuous and dynamic evolution in this field [3]. A significant number of individuals with chronic kidney disease (CKD) in the United States are using NSAIDs, with an observed prevalence of 8% for stages 1 and 2 and 9% for stages 3 and 4, in contrast to patients without CKD, where the prevalence was 5% [4]. In Saudi Arabia, diclofenac, an NSAID, is the most frequently utilized medication, accounting for 67% of all medicines used in the country from 2010 to 2015 [5].

NSAIDs effectively treat a range of conditions, such as osteoarthritis, rheumatoid arthritis, ankylosing spondylitis, menstrual pain, and postoperative discomfort [6]. Furthermore, they are under scrutiny for their potential protective roles in combating significant diseases like cancer and cardiovascular disorders [7]. Moreover, earlier studies demonstrated the potential advantages of NSAIDs in reducing the likelihood of delirium and mortality [8]. Despite the significant progress made over the past two decades, researchers continue to seek methods to design and develop more effective and safer therapies for treating inflammatory conditions [9]. This quest has become particularly compelling due to recognizing inflammation as a shared underlying factor and a unifying mechanism in most chronic diseases [9]. However, the ubiquitous nature of NSAIDs and their availability OTC has led to their frequent use without strict medical supervision. This widespread accessibility and usage have raised concerns regarding the knowledge and awareness of the general population regarding the safe and appropriate utilization of NSAIDs. Nevertheless, NSAIDs come with potential negative impacts on the gastrointestinal (GI) [10], cardiovascular (CV) [11], and renal systems [12]. Additionally, the likelihood of GI bleeding rises with advancing age and in individuals with a history of stomach ulcers and bleeding disorders [13]. For instance, in the USA, NSAID usage among CKD patients raises concerns about the potential for nephrotoxicity and the progression of CKD due to insufficient knowledge regarding the safe use of NSAIDs [14]. Similarly, in Saudi Arabia, the population that used NSAIDs encountered adverse effects associated with these medications. Among these complications, peptic ulcer disease and heartburn emerged as the most prevalent issues [15]. Therefore, it is crucial to explore the level of awareness and understanding the general population possesses about these medications and their associated risks. This multifaceted exploration encompasses a comprehensive assessment of the general public’s knowledge, attitudes, and behaviors concerning NSAID use, focusing on the potential gaps in awareness that could ultimately impact their health and well-being [16].

The knowledge and awareness of the general population regarding NSAID use are critical factors in ensuring safe and responsible medication practices [17]. Understanding appropriate dosages, potential side effects, and interactions with other medications or medical conditions is vital for informed decision-making [18]. Moreover, being aware of alternative pain management strategies and when to seek medical advice can contribute to better health outcomes and reduce the risk of adverse effects associated with NSAIDs. Various factors, including the accessibility of information, cultural norms, and individual beliefs, influence the general population’s awareness of NSAIDs [19]. In an era of readily available information through the internet and media, many individuals rely on online sources and self-diagnosis for their healthcare decisions. This can be both advantageous and risky, as misinformation or misinterpretation of medical information can lead to inappropriate NSAID use or avoidance of necessary treatments [20]. Thus, promoting accurate, evidence-based information on NSAID use and its potential risks is essential in today’s information age.

The utilization of NSAIDs and the awareness of the general population about their use represent a critically important area of study, particularly within the context of Ha’il Province. NSAIDs are a class of medications widely employed for their pain-relieving and anti-inflammatory properties, making them some of the most commonly used pharmaceuticals worldwide. They are easily accessible over the counter in many regions, making them a go-to choice for managing various types of pain and inflammation, from mild headaches to chronic arthritic conditions. However, the benefits of NSAIDs often come with a trade-off, as their use is associated with a range of potential side effects and risks, particularly when taken inappropriately or for extended periods. Understanding the patterns of NSAID utilization within the specific regional context of Ha’il Province is essential to comprehending the broader implications for public health, as usage trends and associated risks can vary significantly from one area to another due to cultural, social, and economic factors. Thus, the present study was designed to investigate the use and knowledge regarding NSAIDs.

## 2. Materials and Methods

### 2.1. Study Design, Sampling, and Data Collection

A cross-sectional study was conducted within the municipality of Hail in Saudi Arabia. This study utilized an online questionnaire (see Supplementary Materials) administered between August and September 2022 to assess the level of awareness and knowledge among the Hail population regarding the appropriate utilization of NSAIDs and the associated complications. Multiple social media platforms, including Twitter, Snapchat, WhatsApp, and Instagram, were employed as dissemination channels for our questionnaire to reach the target population.

### 2.2. Questionnaire Description

The questionnaire was structured into five distinct sections comprising 25 questions. The website that we used for the questionnaire enabled us to detect the IP address/current location/phone that was used and the version of the software. So, matching or duplicate data according to the previous context were excluded. The initial section pertains to collecting demographic data from the study participants, encompassing age, gender, socioeconomic status, marital status, education level, and occupation. The second section was dedicated to eliciting the medical history of the participants, including inquiries related to any chronic diseases they may have experienced, occurrences of GI upset, and the frequency of pain episodes they have encountered. The third section focused on discerning the specific types of NSAIDs employed by the participants. In the fourth section, participants were probed about their NSAID usage habits, encompassing aspects such as the rationale behind their NSAID utilization, the timing of NSAID ingestion, the duration over which these medications were taken, the number of tablets consumed per day, and the sources from which they acquire information about NSAIDs. The fifth and final section of the questionnaire was dedicated to evaluating the knowledge possessed by the participants regarding potential complications associated with NSAID usage.

### 2.3. Pilot Study

The validity and accuracy of the questionnaire were rigorously assessed through a pilot study, ensuring that the instrument was reliable and effectively captured the intended information for the research. The tool used in this current research was a questionnaire developed by referring to standard published literature. The questionnaire was validated with initial responses from the volunteers through Cronbach’s alpha value analysis and Bartlett’s test of sphericity. The questionnaire was validated with an initial 50 responses from the consumer through Cronbach’s alpha value analysis with a value of 0.88 and Bartlett’s test of sphericity (*p* < 0.001).

### 2.4. Sample Size Calculation

We employed the Raosoft^®^ sample size calculator, with a specified margin of error of 5% and a confidence level set at 95%, and, after calculations, a 377 sample size was obtained; furthermore, sampling error was taken into account by calculating 377 × 1.058, which corresponded to 399.

### 2.5. Statistical Analysis

The data collected were subjected to encoding and preprocessing procedures before statistical analysis, which involved employing MS Excel for data manipulation and organization. Subsequent statistical analyses were performed using SPSS-21 software version 21. Descriptive statistical metrics, specifically frequencies and proportions represented as percentages, were computed to examine an array of qualitative variables encompassing socio-demographic attributes and factors pertaining to NSAID utilization. Chi-square test was used to see the association between reason for using NSAIDs and different groups (demographics). Logistic regression analyses were performed to identify the factors significantly associated with NSAID usage. A *p* value of <0.05 was considered statistically significant throughout the analyses. In the subsequent multiple logistic regression analysis, independent variables were subjected to a rigorous selection process, employing a significance threshold set at a *p*-value < 0.05 for screening and inclusion.

## 3. Results

### 3.1. Demographic Characteristics

The gender distribution indicated that 170 participants (42.61%) were male, whereas 229 (57.39%) were female. In terms of age, 73 participants (18.43%) fell within the 18–20 age range, 124 (30.96%) were between the ages of 21–29, 54 (13.51%) were aged 30–39, and 85 (21.13%) were in the 40–49 age group. Education levels varied, with 79 participants (19.9%) having completed high school or less, 44 (11.12%) with a diploma, 261 (65.6%) holding a bachelor’s degree, 18 (4.45%) having a master’s degree, and 9 (2.36%) possessing a PhD. In terms of occupation, 26 participants (6.52%) worked in the private sector, 123 (30.83%) held government jobs, 12 (3.01%) were involved in business, 42 (10.53%) were housewives, 25 (6.27%) were unemployed, 24 (6.02%) were retired, 3 (1%) worked from home, and 143 (35.84%) were students. Regarding monthly income, 133 participants (33.34%) reported earnings above the average, 195 (48.9%) had an average income, and 73 (18.3%) earned below the average. Marital status revealed that 7 participants (1.8%) were divorced, 202 (50.6%) were married, 188 (47.1%) were single, and 2 (0.5%) were widowed (Table 1). In terms of reasons, muscle pain was reported by 53 (13.34%) participants, whereas headaches were a concern for 109 (27.4%) participants. Fever was experienced by 33 (8.23%) participants, and toothache affected 68 (17.2%) participants. Menstrual cycle-related issues were reported by 60 (14.92%) participants, and joint pain was observed in 52 (13.2%) of the cases. The remaining health issues, categorized as “Others”, were reported by 20 (5.1%) of the cases (Table 1).

Additionally, association between demographic variables and reasons for using NSAIDs, such as muscle pain, headache, fever, toothache, menstrual cycle, joint pain, and others, were presented with associated *p*-values, indicating the level of significance. Gender, occupation, and marital status had non-significant association (*p* > 0.01), except for menstrual cycle and joint pain, whereas marital status also had significant association with other reasons. Age had significant association with muscle pain, menstrual cycle, and joint pain (*p* < 0.01). Meanwhile, education and monthly income had non-significant associations with all the reasons (Table 2).

### 3.2. Association between Demographic Variables and Pain Frequency

In terms of gender, a significant association (*p* = 0.01) was found, as 99 males (47.6%) experienced irregular pain, 7 (30.4%) had daily pain, 25 (53.2%) reported weekly pain, and 39 (32.2%) experienced monthly pain. Among females, 109 (52.4%) experienced irregular pain, 16 (69.6%) had daily pain, 22 (46.8%) reported weekly pain, and 82 (67.8%) experienced monthly pain. Education level also played a significant role, with participants with a high school education or less having 39 participants (18.8%) experiencing irregular pain, 5 (21.7%) having daily pain, 13 (27.7%) having weekly pain, and 20 (16.5%) experiencing monthly pain. Lastly, participants with a PhD had 2 (1.0%) experiencing irregular pain, 3 (13.0%) with daily pain, 2 (4.3%) with weekly pain, and none with monthly pain, with a significant association (*p* = 0.001) (Table 3).

In Table 4, the regression analysis revealed that gender played a significant role, with females having higher odds of knowledge (AOR = 1.75, 95% CI 1.10–2.88) than males. Age also had a notable impact, with individuals aged 50–59 (AOR = 0.21, 95% CI 0.07–0.66) and those above 60 (AOR = 0.30, 95% CI 0.05–1.92) showing lower odds of knowledge compared to the reference group (18–20 years). Education had varied effects, where individuals with a PhD (AOR = 3.50, 95% CI 0.54–22.51) exhibited the highest odds of knowledge among the education categories. Occupation-wise, those in business (AOR = 6.12, 95% CI 1.10–35.10) had significantly higher odds of knowledge than those in the private sector, whereas housewives (AOR = 0.76, 95% CI 0.25–2.27) had lower odds. Monthly income did not show significant associations with knowledge.

In Table 5, the bifurcation of medications concerning gender revealed differences in the use of various medications between females and males. When it came to aspirin, 202 females (56.6%) answered “No”, whereas 27 females (64.3%) answered “Yes”, compared to 155 males (43.4%) answering “No” and 15 males (35.7%) answering “Yes”, with a *p*-value of 0.34. For diclofenac, 110 females (50.2%) answered “No”, and 119 females (66.1%) answered “Yes”, whereas 109 males (49.8%) answered “No”, and 61 males (33.9%) answered “Yes”, with a significant *p*-value of 0.001. Similar differences were observed with ibuprofen (*p*-value = 0.01), celecoxib (*p*-value = 0.004), and mefenamic acid (*p*-value = 0.08). However, ketoprofen, meloxicam, naproxen, and piroxicam did not show statistically significant differences between the genders, with *p*-values of 0.58, 0.76, 1.00, and 0.36, respectively.

**Figure 1:**
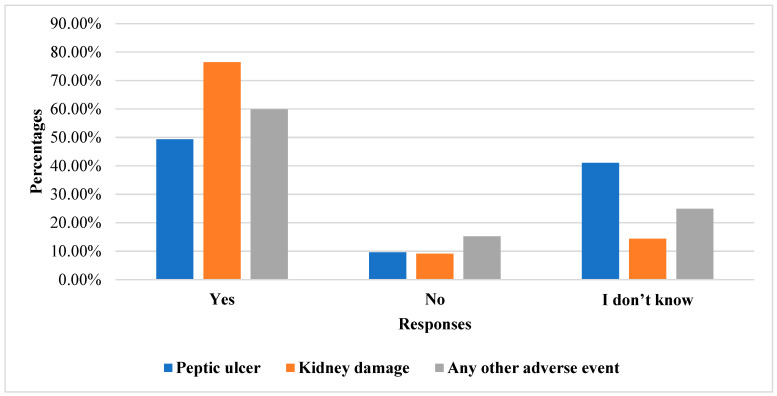
Knowledge of adverse events related to the use of NSAIDs.

Meanwhile, >50% of the participants had knowledge of adverse events related to the use of NSAIDs, whereas 25% had no knowledge. Moreover, 59 (25.76%) participants reported discomfort with the use of NSAIDs. In addition, 50% and >75% of participants’ thought NSAIDs could cause peptic ulcers and damage kidneys, respectively (Figure 1). Meanwhile, 39.3% of participants wanted all NSAIDs to be available without prescription.

In the present study, it was found that regarding the sources of information about the use of NSAIDs concerning gender, several significant differences were observed. Regarding information from physicians, a *p*-value of 0.12 indicated non-statistically significant difference between females and males, with 75.5% of females and 81.8% of males responding negatively. In comparison, 24.5% of females and 18.2% of males responded positively. However, information from pharmacists yielded a notable difference with a *p*-value of 0.01, suggesting that more females (69.4%) received information negatively compared to males (80.6%), where 30.6% of females and 19.4% of males received positive information. Information from relatives also showed a significant disparity with a *p*-value of 0.001, as 82.1% of females and 94.1% of males did not receive information from relatives. In comparison, 17.9% of females and 5.9% of males did. Information from friends, social media, and other sources did not exhibit significant differences between females and males, as indicated by their *p*-values of 0.86, 0.13, and 0.60. Moreover, the frequency of NSAID usage revealed a significant discrepancy with a *p*-value of 0.001, demonstrating that more females (33.2%) did not use the tablets compared to males (55.3%), and the usage pattern varied across the categories (Table 6). Use of various NSAIDs with respect to gender is presented in (Table 7).

## 4. Discussion

NSAIDs are among the most commonly used medications due to their anti-inflammatory, anti-pyretic, and analgesic properties. However, improper use of NSAIDs in the long term is associated with GI, cardiovascular, and renal toxicity. The widespread use of NSAIDs worldwide and their serious adverse effects cause the severe problem of NSAIDs [3]. This study was the first to assess the people’s awareness of NSAIDs use in the Hail region, Saudi Arabia.

In the present study, age, gender, occupation, and marital status had a significant association (*p* < 0.01) with menstrual cycle and joint pain, whereas age also had significant association with muscle pain. The significant associations between age, gender, occupation, and marital status with menstrual cycle and joint pain could be attributed to various factors. Age, being a significant factor in the use of NSAIDs for muscle pain, might suggest that as individuals age, they are more likely to experience muscle pain, thereby leading to NSAID use and most used drugs in elderly people [21]. Gender differences may be linked to variations in pain thresholds and reporting, with women potentially experiencing menstrual cycle and joint pain more frequently [22]. Occupational and marital status may influence the physical demands of a person’s work and their social support network, which, in turn, can impact the experience of joint pain. These findings underscore the complex interplay of demographic variables in shaping individuals’ pain experiences and subsequent use of NSAIDs. Moreover, it was also observed that 57.4% of the participants had utilized NSAIDs, signifying that NSAID usage within the Hail region is prevalent and readily accessible. Our study further revealed that the predominant NSAID used was diclofenac, which aligns with prior research conducted in Saudi Arabia, where diclofenac was reported as the most frequently used drug within the country across all drug classes [7]. Additionally, consistent with findings from a study in Turkey, diclofenac emerged as the most commonly used NSAID [23]. In contrast, a study conducted in Saudi Arabia found that the prevalent type of NSAID was ibuprofen [15]. These observations underscore the variability in NSAID utilization patterns, which can be attributed to regional and cultural distinctions in drug preferences.

Our study shows that a notable proportion of participants use NSAIDs without a prescription, reflecting a relatively permissive attitude toward their utilization, and 39.3% of participants believed in the accessibility of NSAIDs without any prescription. Notably, this finding aligns with the opinions of around one-third of the participants, who opined that such medications should be available without needing a prescription. Similarly, without a prescription, NSAIDs were taken by 83.7% of medical students and 84.7% of non-medical students in Karachi, Pakistan [24].

Furthermore, our results indicate that a substantial proportion of NSAID users, approximately >50%, reported an absence of discomfort, side effects, or problems associated with their usage. While approximately 50% of participants reported a history of GI upset (peptic ulcer), it remains challenging to conclusively attribute this to using NSAIDs alone, as other contributing factors may be at play. Moreover, a breakdown of NSAID usage patterns revealed that about 62% of participants employed NSAIDs for 1 to 3 days, whereas roughly 22% extended usage to 4 to 7 days. Of note, it is imperative to acknowledge that even from the inception of use, all NSAIDs engender an elevated risk of adverse effects, including GI bleeding, myocardial infarction, and stroke [25,26]. Notably, NSAIDs are associated with a rise in systolic blood pressure by approximately 5 mmHg and an increase in fluid retention. In patients administered coxibs, diclofenac, and higher-dose ibuprofen, these effects contribute to an excess risk of approximately 7 to 9 non-fatal and 2 fatal cardiovascular events per 1000 patients annually. This underlines the heightened risk of developing adverse effects due to NSAIDs from the very onset of usage [2].

Our study also revealed that the participants (male and female) sourced information about NSAIDs from physicians and pharmacists, indicating the significant role played by pharmacists in patient counseling. This observation aligns with the findings of two studies conducted in Saudi Arabia [15]. Concerning perceptions of NSAIDs, over 60% of the participants believed that NSAIDs could lead to developing diseases, whereas around 25% perceived them as not causing any diseases. A notable percentage, approximately 41%, expressed uncertainty regarding the relationship between NSAIDs and peptic ulcers, with roughly 10% of participants asserting that NSAIDs did not cause peptic ulcers at all. These findings underscore a lack of awareness among nearly half of the participants regarding the gastrointestinal complications associated with NSAIDs, which may potentially progress to more severe conditions, such as cancer. Furthermore, our study indicates that about 77% of participants concurred that NSAIDs could contribute to kidney damage. Notably, almost half of the participants admitted to being uninformed about the potential side effects of NSAIDs, highlighting a significant knowledge gap concerning the adverse effects of NSAIDs, which can result in severe systemic repercussions. These findings align with a study conducted in Poland, which reported that approximately 57% of individuals were unaware of the side effects associated with NSAIDs [27]. One limitation of our research is that the sample size was restricted to a single region, and there was no evaluation of the potential side effects of individual class or specific NSAIDs.

## 5. Conclusions

The findings of our investigation underscore a notable deficiency in the participants’ understanding of NSAIDs usage and its associated complications. This highlights a pressing imperative for comprehensive public education initiatives in several respects. Enhancing public awareness concerning alternative pharmaceutical options characterized by comparably reduced adverse effects relative to widely utilized drugs is imperative. Furthermore, healthcare practitioners are pivotal in disseminating pertinent information regarding medication usage and its attendant implications to patients. Community pharmacists, in particular, are responsible for providing guidance to individuals procuring medications without prior medical consultation. The discerned inadequacy concerning NSAID utilization and associated risks necessitates immediate and extensive public enlightenment efforts to promote judicious NSAID utilization.

## Figures and Tables

**Table 1:** Demographic variable of the respondents (*n* = 399).

Demographic	Variables	Number	Percentage
Gender	Male	170	42.61
	Female	229	57.39
Age	18–20	73	18.43
	21–29	124	30.96
	30–39	54	13.51
	40–49	85	21.13
Education	High School or Less	79	19.9
	Diploma	44	11.12
	Bachelors	261	65.6
	Master	18	4.45
	PhD	9	2.36
Occupation	Private Sector	26	6.52
	Government Job	123	30.83
	Business	12	3.01
	House Wife	42	10.53
	Unemployed	25	6.27
	Retired	24	6.02
	Work From Home	3	1
	Student	143	35.84
Monthly Income	Above the Average	133	33.34
	Average	195	48.9
	Below the Average	73	18.3
Marital status	Divorced	7	1.8
	Married	202	50.6
	Single	188	47.1
	Widowed	2	0.5
Reasons for use	Muscle Pain	53	13.34
	Headache	109	27.4
	Fever	33	8.23
	Toothache	68	17.2
	Menstrual Cycle	60	26.20 *
	Joint Pain	52	13.2
	Others	20	5.1

* Percentage of pain due to menstrual cycle is calculated only for female participants.

**Table 2:** Association between demographic variables and reasons for using NSAIDs (*n* = 399).

Demographic Factors						
Variables	Gender *p*-Value	Age *p*-Value	Education *p*-Value	Occupation *p*-Value	Monthly Income *p*-Value	Marital Status *p*-Value
Muscle pain	0.34	0.01	0.24	0.12	0.55	0.14
Headache	0.11	0.31	0.19	0.85	0.18	0.18
Fever	0.16	0.03	0.63	0.26	0.34	0.53
Toothache	0.10	0.35	0.12	0.11	0.22	0.66
Menstrual cycle	0.001	0.001	0.23	0.01	0.05	0.01
Joint pain	0.002	0.001	0.05	0.001	0.52	0.001
Others	0.29	0.18	0.45	0.25	0.67	0.01

Chi-square test was used to see the association between reason for using NSAIDs and different groups (demographics). A *p* value of <0.05 was considered statistically significant throughout the analyses.

**Table 3:** Association between demographic variable and pain frequency.

Variables	Categories	Pain Frequency				
		Irregular	Daily	Weekly	Monthly	*p*-Value
Gender	Male	99 (47.6)	7 (30.4)	25 (53.2)	39 (32.2)	0.01
	Female	109 (52.4)	16 (69.6)	22 (46.8)	82 (67.8)	
Age (years)	18–20	30 (14.4)	5 (21.7)	14 (29.8)	26 (21.5)	0.03
	21–29	67 (32.2)	5 (21.7)	12 (25.5)	42 (34.7)	
	30–39	29 (13.9)	6 (26.1)	4 (8.5)	16 (13.2)	
	40–49	46 (22.1)	2 (8.7)	7 (14.9)	31 (25.6)	
	50–59	30 (14.4)	4 (17.4)	8 (17.0)	4 (3.3)	
	Above 60	6 (2.9)	1 (4.3)	2 (4.3)	2 (1.7)	
Education	High School or Less	39 (18.8)	5 (21.7)	13 (27.7)	20 (16.5)	0.001
	Diploma	25 (12.0)	2 (8.7)	5 (10.6)	12 (9.9)	
	Bachelors	136 (65.4)	10 (43.5)	26 (55.3)	85 (70.2)	
	Master	6 (2.9)	3 (13.0)	1 (2.1)	4 (3.3)	
	PHD	2 (1.0)	3 (13.0)	2 (4.3)	-	
Occupation	Private Sector	14 (6.7)	-	3 (6.4)	9 (7.4)	0.43
	Government Job	68 (32.7)	8 (34.8)	14 (29.8)	33 (27.3)	
	Business	2 (1.0)	1 (4.3)	2 (4.3)	7 (5.8)	
	House Wife	25 (12.0)	3 (13.0)	4 (8.5)	10 (8.3)	
	Unemployed	13 (6.3)	2 (8.7)	-	10 (8.3)	
	Retired	14 (6.7)	2 (8.7)	4 (8.5)	4 (3.3)	
	Work From Home	1 (0.5)	-	-	3 (2.5)	
	Student	71 (34.1)	7 (30.4)	20 (42.6)	45 (37.2)	
Monthly Income	Above the Average	63 (30.3)	8 (34.8)	23 (48.9)	36 (29.8)	0.12
	Average	101 (48.6)	9 (39.1)	19 (40.4)	66 (54.5)	
	Below the Average	44 (21.2)	6 (26.1)	5 (10.6)	19 (15.7)	
Marital status	Divorced	2 (1.0)	-	-	5 (4.1)	0.20
	Married	111 (53.4)	12 (52.2)	22 (46.8)	57 (47.1)	
	Single	95 (45.7)	10 (43.5)	25 (53.2)	58 (47.9)	
	Widowed	-	1 (4.3)	-	1 (0.8)	

Regression analysis for the association between pain frequency and different groups (demographics). A *p* value of <0.05 was considered statistically significant.

**Table 4:** Regression model for demographic variables and knowledge (*n* = 399).

Variables	Categories	Regression Model	
		COR (95% CI)	AOR (95% CI)
Gender	Male	1	1
	Female	1.53 (1.10, 2.30)	1.75 (1.10, 2.88)
Age (years)	18–20	1	1
	21–29	0.60 (0.33, 1.05)	0.68 (0.34, 1.37)
	30–39	0.59 (0.29, 1.21)	0.50 (0.17, 1.34)
	40–49	0.58 (0.31, 1.08)	0.39 (0.15, 1.05)
	50–59	0.36 (0.17, 0.76)	0.21 (0.07, 0.66)
	Above 60	0.56 (0.15, 1.98)	0.30 (0.05, 1.92)
Education	High School or Less	1	1
	Diploma	0.78 (0.37, 1.64)	0.94 (0.40, 2.22)
	Bachelors	0.91 (0.43, 4.32)	0.92 (0.52, 1.64)
	Master	1.37 (0.43, 4.32)	1.42 (0.41, 4.94)
	PHD	2.60 (0.46, 14.04)	3.50 (0.54, 22.51)
Occupation	Private Sector	1	1
	Government Job	0.94 (0.40, 2.21)	1.15 (0.44, 2.97)
	Business	5.83 (1.06, 32.02)	6.12 (1.10, 35.10)
	House Wife	0.79 (0.30, 2.13)	0.76 (0.25, 2.27)
	Unemployed	1.10 (0.35, 3.23)	1.16 (0.37, 3.63)
	Retired	0.83 (0.27, 2.55)	1.52 (0.35, 6.45)
	Work From Home	3.50 (0.32, 38.23)	3.53 (0.29, 42.70)
	Student	1.20 (0.51, 2.73)	0.76 (0.29, 1.97)
Monthly Income	Above the Average	1	1
	Average	0.87 (0.56, 1.36)	0.77 (0.47, 1.25)
	Below the Average	0.64 (0.36, 1.14)	0.65 (0.34, 1.22)

**Table 5:** Bifurcation of medications with respect to gender.

Variables	Categories	Regression Model	
		COR (95% CI)	AOR (95% CI)
Gender	Male	1	1
	Female	1.53 (1.10, 2.30)	1.75 (1.10, 2.88)
Age (years)	18–20	1	1
	21–29	0.60 (0.33, 1.05)	0.68 (0.34, 1.37)
	30–39	0.59 (0.29, 1.21)	0.50 (0.17, 1.34)
	40–49	0.58 (0.31, 1.08)	0.39 (0.15, 1.05)
	50–59	0.36 (0.17, 0.76)	0.21 (0.07, 0.66)
	Above 60	0.56 (0.15, 1.98)	0.30 (0.05, 1.92)
Education	High School or Less	1	1
	Diploma	0.78 (0.37, 1.64)	0.94 (0.40, 2.22)
	Bachelors	0.91 (0.43, 4.32)	0.92 (0.52, 1.64)
	Master	1.37 (0.43, 4.32)	1.42 (0.41, 4.94)
	PHD	2.60 (0.46, 14.04)	3.50 (0.54, 22.51)
Occupation	Private Sector	1	1
	Government Job	0.94 (0.40, 2.21)	1.15 (0.44, 2.97)
	Business	5.83 (1.06, 32.02)	6.12 (1.10, 35.10)
	House Wife	0.79 (0.30, 2.13)	0.76 (0.25, 2.27)
	Unemployed	1.10 (0.35, 3.23)	1.16 (0.37, 3.63)
	Retired	0.83 (0.27, 2.55)	1.52 (0.35, 6.45)
	Work From Home	3.50 (0.32, 38.23)	3.53 (0.29, 42.70)
	Student	1.20 (0.51, 2.73)	0.76 (0.29, 1.97)
Monthly Income	Above the Average	1	1
	Average	0.87 (0.56, 1.36)	0.77 (0.47, 1.25)
	Below the Average	0.64 (0.36, 1.14)	0.65 (0.34, 1.22)

**Table 6:** Bifurcation of sources of information about the use of non-steroidal anti-inflammatory drugs, with respect to gender.

Variables	Categories	Female	Male	*p*-Value
Physician				
	No	173 (75.5)	139 (81.8)	0.12
	Yes	56 (24.5)	31 (18.2)	
Pharmacist				
	No	159 (69.4)	137 (80.6)	0.01
	Yes	70 (30.6)	33 (19.4)	
Relatives				
	No	188 (82.1)	160 (94.1)	0.001
	Yes	41 (17.9)	10 (5.9)	
Friends				
	No	209 (91.3)	156 (91.8)	0.86
	Yes	20 (8.7)	14 (8.2)	
Social media				
	No	213 (93.0)	164 (96.5)	0.13
	Yes	16 (7.0)	6 (3.5)	
Others				
	No	217 (94.8)	159 (93.5)	0.60
	Yes	12 (5.2)	11 (6.5)	
Frequency				
	Not used	76 (33.2)	94 (55.3)	0.001
	1	82 (35.8)	42 (24.7)	
	2	49 (21.4)	25 (14.7)	
	3	16 (7.0)	6 (3.5)	
	4	2 (0.9)	1 (0.6)	
	More than 4 tablets	4 (1.7)	2 (1.2)	

Regression analysis for the association between NSAID use with respect to gender. A *p* value of <0.05 was considered statistically significant.

**Table 7:** Bifurcation of NSAIDs with respect to sex.

Variables	Categories	Female	Male	*p*-Value
Aspirin	No	202 (56.6)	155 (43.4)	0.34
	Yes	27 (64.3)	15 (35.7)	
Diclofenac	No	110 (50.2)	109 (49.8)	0.001
	Yes	119 (66.1)	61 (33.9)	
Ibuprofen	No	160 (53.7)	138 (46.3)	0.01
	Yes	69 (69.0)	32 (31.0)	
Celecoxib	No	205 (55.4)	165 (44.6)	0.004
	Yes	24 (82.8)	5 (17.2)	
Ketoprofen	No	228 (57.6)	168 (42.4)	0.58
	Yes	1 (33.3)	2 (66.7)	
Mefenamic acid	No	222 (56.8)	169 (43.2)	0.08
	Yes	7 (87.5)	1 (12.5)	
Meloxicam	No	222 (56.8)	166 (42.8)	0.76
	Yes	7 (87.5)	4 (36.4)	
Naproxen	No	223 (57.3)	166 (42.7)	1.00
	Yes	6 (60.0)	4 (40.0)	
Piroxicam	No	221 (57.0)	167 (43.0)	0.36
	Yes	8 (72.7)	3 (27.3)	

Regression analysis for the association between NSAID use with respect to sex. A *p* value of <0.05 was considered statistically significant.

## Data Availability

Data are contained within the article.

## Supplementary Materials

The following supporting information can be downloaded at: https://www.mdpi.com/article/10.3390/pharmacy12010009/s1.
