# Supplementary material for: Charting the Knowledge and Patterns of Non-Steroidal Anti-Inflammatory Drugs Usage in Hail Population, Saudi Arabia: Insights into the Adverse Effect Profile
Source: Pharmacy (Basel). 2024 Jan 8;12(1):9. doi: 10.3390/pharmacy12010009 (PMC10801559; doi:10.3390/pharmacy12010009)
Supplement: Supplementary file 1 [file pharmacy-12-00009-s001.zip › pharmacy-2769212-supplementary.pdf]

Link for survey: https://sprw.io/stt-9e6dd9

سؤال 1

القسم الأول

الرجاء الإجابة على الأسئلة التالية (خذ وقتك في الإجابة على الأسئلة)

سؤال 1.1

عمرك؟

- 17 سنة او اقل
- 18 الى 20 سنة
- 21 الى 29 سنة
- 30 الى 39 سنة
- 40 الى 49 سنة
- 50 الى 59 سنة
- 60 سنة او اكبر

سؤال 1.2

جنسك؟

- ذكر
- انثى

سؤال 1.3

الحالة المادية ؟

وفقاً للهيئة العامة للأحصاء لعام 2018 ميلادي متوسط الدخل في حائل للأسرة يبلغ 9,701 ريال سعودي

- على من المتوسط
- متوسط

ا

سؤال 1.4

حالتك الاجتماعية؟

- اعزب
- متزوج / متزوجة
- منفصل
شريك

سؤال 1.5

مستوى التعليم؟

- الثانوية العامة او اقل
- دبلوم
- بكالوريوس
- ماجستير
- دكتوراة

سؤال 1.6

مهنتك؟

- قطاع خاص
- وظيفة حكومية
- اعمال تجارية
- ربة منزل
- عاطل عن العمل
- متقاعد
- العمل من المنزل
- طالب

سؤال 2

القسم الثاني

الرجاء الإجابة على الأسئلة التالية (خذ وقتك في الإجابة على الأسئلة)

سؤال 2.1

وتيرة تكرر الألم لديك؟

جميع أنواع الألم مثل صداع, ألم دورة شهرية, ألم أسنان غير ذلك

- شهريا
- أسبوعيا
- يوميا
- غير منتظم

سؤال 2.2

الأمراض المزمنة التي تعاني منها؟

(اختيار متعدد)
امثله على امراض القلب : السكتة الدماغية , الذبحة الصدرية, امراض القلب التاجية, فشل القلب الاحتقاني وغير ذلك

- ارتفاع ضغط الدم
- داء السكري
- الربو
- الفشل الكلوي
- امراض القلب
- الم المفاصل
- لا يوجد أي امراض مزمنة
- غير ذلك

سؤال 2.3

هل تعاني اضطرابات في الجهاز الهضمي؟

- نعم
- لا

سؤال 3

القسم الثالث

هل تستخدم إحدى مضادات الالتهاب غير الستيرويدية (مسكنات الألم) التالية؟

هل تستخدم إحدى مضادات الالتهاب غير الستيرويدية (مسكنات الألم) التالية؟

أسبرين

سؤال 3.2

هل تستخدم إحدى مضادات الالتهاب غير الستيرويدية (مسكنات الألم) التالية؟

ديكلوفيناك

ملحوظة: يوجد عدة صور وجميع صور لعدة شركات تجارية لكن تحتوي على نفس المادة الفعالة

سؤال 3.3

هل تستخدم إحدى مضادات الالتهاب غير الستيرويدية (مسكنات الألم) التالية؟

ايبوبروفين

ملحوظة: يوجد عدة صور وجميع صور لعدة شركات تجارية لكن تحتوي على نفس المادة الفعالة

سؤال 3.4

مضادات الالتهاب غير الستيرويدية (مسكنات الألم) الآخر

اختار أي من الأدوية التالية التي قد تم استخدامها

(اختيار متعدد)

ملحوظة: يوجد عدة صور وجميع صور لعدة شركات تجارية لكن تحتوي على نفس المادة الفعالة

سؤال 4

القسم الرابع

الرجاء الإجابة على الأسئلة التالية (خذ وقتك في الإجابة على الأسئلة)

سؤال 4.1

وقت تناولك مضادات الالتهاب غير الستيرويدية (مسكنات الألم)؟

المضادات الالتهاب غير الستيروئيدية هي جميع ما تم عرضة أو ذكره في القسم الثالث.

- قبل الوجبة
- بعد الوجبة
- اثناء الوجبة
- أي وقت

سؤال 4.2

عدد حبات مضادات الالتهاب غير الستيرويدية (مسكنات الألم) التي تم استخدامها في اليوم؟

المضادات الالتهاب غير الستيروئيدية هي جميع ما تم عرضة أو ذكره في القسم الثالث.

- 1
- 2
- 3
- 4
- اكثر من 4 حبات

سؤال 4.3

عدد أيام استخدامك مضادات الالتهاب غير الستيرويدية (مسكنات الألم)؟

المضادات الالتهاب غير الستيروئيدية هي جميع ما تم عرضة أو ذكره في القسم الثالث.

- 1 الى ثلاث أيام
- 4 الى 7 أيام
- اكثر من 14 يوم

سؤال 4.4

سبب أو أسباب استخدامك مضادات الالتهاب غير الستيرويدية (مسكنات الألم)؟

المضادات الالتهاب غير الستيروئيدية هي جميع ما تم عرضة أو ذكره في القسم الثالث.
(اختيار متعدد)

- لام العضلات
- صداع
- الحمى
- الم الاسنان
- دورة شهرية
- الم مفاصل
- غير ذلك

سؤال 4.5

هل عانيت من أي إزعاج أو أثار جانبية أو مشكلة بسبب استخدامك مضادات الالتهاب غير الستيرويدية (مسكنات الألم)؟

المضادات الالتهاب غير الستيروئيدية هي جميع ما تم عرضة أو ذكره في القسم الثالث.
من الأعراض المحتملة: عسر الهضم - فرط الحساسية - إرتفاع ضغط الدم - إمساك - غثيان وقيء - ضيق التنفس.

- نعم
- لا

مصدر المعلومات حول استخدامك مضادات الالتهاب غير الستيرويدية (مسكنات الألم)؟

المضادات الالتهاب غير الستيروئيدية هي جميع ما تم عرضة أو ذكره في القسم الثالث.

- طبيب
- صيدلي
- الاقرباء
- الأصدقاء
- وسائل التواصل الاجتماعي
- غير ذلك

سؤال 5

القسم الخامس

الرجاء الإجابة على الأسئلة التالية (خذ وقتك في الإجابة على الأسئلة)

سؤال 5.1

هل تعتقد أن جميع الأدوية من مجموعة مضادات الالتهاب غير الستيرويدية (مسكنات الألم) يجب أن تباع بدون وصفة طبية؟

المضادات الالتهاب غير الستيروئيدية هي جميع ما تم عرضة أو ذكره في القسم الثالث.

- نعم
- لا
- لا اعلم

سؤال 5.2

هل تعتقد أن مضادات الالتهاب غير الستيرويدية (مسكنات الألم) يمكن أن تسبب أي مرض؟

المضادات الالتهاب غير الستيروئيدية هي جميع ما تم عرضة أو ذكره في القسم الثالث.

- نعم
- لا
- لا اعلم

سؤال 5.3

هل يمكن أن مضادات الالتهاب غير الستيرويدية (مسكنات الألم) يمكن أن تسبب أمراض القرحة الهضمية (النزيف)؟

المضادات الالتهاب غير الستيروئيدية هي جميع ما تم عرضة أو ذكره في القسم الثالث.

- نعم
- لا
- لا اعلم

سؤال 5.4

هل تعتقد أن مضادات الالتهاب غير الستيرويدية (مسكنات الألم) يمكن أن تسبب اضرار بالكلى؟

المضادات الالتهاب غير الستيروئيدية هي جميع ما تم عرضة أو ذكره في القسم الثالث.

- نعم
- لا
- لا اعلم

سؤال 5.5

هل تعرف الآثار السلبية لأستخدامك مضادات الالتهاب غير الستيرويدية (مسكنات الألم)؟

المضادات الالتهاب غير الستيروئيدية هي جميع ما تم عرضة أو ذكره في القسم الثالث.

- نعم
- لا
- لا اعلم

List of questionnaires (English)

1. Socioeconomic

i. Age ; ii. Sex; iii. Socioeconomic status; iv.Marital Status; v.Educational Level; vi.Occupation

2. NSAIDS usage

i. Do you use the following non-steroidal anti-inflammatory drugs (pain relievers)?
ii. Pain frequency? (All kinds of pain such as headache, menstrual pain, toothache, etc.)
iii. Monthly; Weekly; Daily; Irregular.
iv. Chronic illness ; Hypertension, Diabetes, Asthma, Renal Failure, Heart disease, Joint pain, No chronic illness, other.
v. History of GI upsets

3.Which NSAIDS used

i. Do you use the following non-steroidal anti-inflammatory drugs (pain relievers)? Aspirin (picture)
ii. Do you use the following non-steroidal anti-inflammatory drugs (pain relievers)? Diclofenac (picture)
iii. Do you use the following non-steroidal anti-inflammatory drugs (pain relievers)? Ibuprofen
iv. Choose any of the following medicines that have been used.

4.NSAIDs and related side-effects

i. When do you take non-steroidal anti-inflammatory drugs (pain relievers)?
ii. How many non-steroidal anti-inflammatory drugs (pain relievers) pills are used per day?
iii. The number of days of use for the non-steroidal anti-inflammatory drugs (pain relievers)?
iv. The reason(s) for using non-steroidal anti-inflammatory (pain relievers) drugs?
v. Have you experienced any discomfort, side effects or problem with non-steroidal anti-inflammatory drugs use (pain relievers)?

5.Non-steroidal anti-inflammatory drugs was shown in third section.

i. Possible symptoms: indigestion - hypersensitivity - high blood pressure - constipation - nausea and vomiting shortness of breath.
ii. Sources of information about the use of Non-steroidal anti-inflammatory drugs (pain relievers)?

6.Do you think that all medication from the NSAIDs drugs group should be sold without a prescription (pain relievers)?

Non-steroidal anti-inflammatory drugs was shown in third section.

NSAIDs : Non-steroidal anti-inflammatory drugs.

i. Do you think that NSAIDs drugs can cause any disease (pain relievers)?
ii. Do you think that NSAIDs drugs (pain relievers) cause peptic ulcer disease (bleeding)?

iii. Do you think NSAIDs (pain relievers) can cause kidney damage?
iv. Do you know the adverse effects of NSAIDs drugs use (pain relievers) ?
